# Universal Oligonucleotide Microarray for Sub-Typing of Influenza A Virus

**DOI:** 10.1371/journal.pone.0017529

**Published:** 2011-04-29

**Authors:** Vladimir A. Ryabinin, Elena V. Kostina, Galiya A. Maksakova, Alexander A. Neverov, Konstantin M. Chumakov, Alexander N. Sinyakov

**Affiliations:** 1 Institute of Chemical Biology and Fundamental Medicine, Siberian Branch, Russian Academy of Sciences, Novosibirsk, Russia; 2 Center of Biologics Evaluation and Research, Food and Drug Administration, Rockville, Maryland, United States of America; University of Georgia, United States of America

## Abstract

A universal microchip was developed for genotyping Influenza A viruses. It contains two sets of oligonucleotide probes allowing viruses to be classified by the subtypes of hemagglutinin (H1–H13, H15, H16) and neuraminidase (N1–N9). Additional sets of probes are used to detect H1N1 swine influenza viruses. Selection of probes was done in two steps. Initially, amino acid sequences specific to each subtype were identified, and then the most specific and representative oligonucleotide probes were selected. Overall, between 19 and 24 probes were used to identify each subtype of hemagglutinin (HA) and neuraminidase (NA). Genotyping included preparation of fluorescently labeled PCR amplicons of influenza virus cDNA and their hybridization to microarrays of specific oligonucleotide probes. Out of 40 samples tested, 36 unambiguously identified HA and NA subtypes of Influenza A virus.

## Introduction

Influenza A virus circulating among humans and domestic and wild animals presents a significant pandemic threat due to the rapid evolution of strains with new antigenic properties [1]. Examples include the recent emergence of H1N1 swine influenza virus that proved pathogenic for humans, as well as H5N1 bird influenza virus that has the capacity to become pathogenic for domestic birds and humans.

Since protection against influenza is determined primarily by HA and NA antigens, it is important to classify new isolates by subtypes of these proteins. Presently, 16 sub-types of HA and 9 sub-types of NA are known [2], [3]. The traditional scheme of sub-typing influenza viruses involves virus isolation, analysis using standard immunochemical techniques or real-time PCR amplification, and partial or complete nucleotide sequencing of HA and NA genes [4], [5], [6], [7].

Recently, microchip hybridization has become a popular diagnostic tool [8], [9], [10], [11], [12], [13], [14], [15], [16], [17], [18], [19], [20], [21], [22]. Two types of microchips are used for this purpose: sequencing and hybridization microchips. In the first case, the use of microchip and micro-fluidics technology is used for determination of nucleotide sequences of HA and NA needed for the classification of viruses [14], . More commonly, hybridization microchips use sets of immobilized oligonucleotide probes that form complexes with virus-specific cDNA tagged with, for instance, a fluorescent label [9], [13], [17], [21], [22]. In these papers specialized microchips were used to determine a limited number of influenza virus types [9], [13], as well as microchips for sub-typing the majority of HA and NA variants [20]. Some protocols use PCR amplification with primers specific to select sub-types of HA and NA [22]. These protocols increase specificity by complicating the overall procedure. Efficiency of any microchip hybridization protocol is determined by the procedure used to identify specific probes to distinguish between genotypes. The task of probe selection is relatively straightforward when sequences differ significantly, as is the case for the genotyping of higher taxa. For instance, it is very easy to discriminate herpes and pox-viruses [23].

The task becomes more complicated if genomic structures are similar. Probe selection becomes even more complicated when the number of known sequences is very large and exceeds several hundreds and even thousands. In this case, the strategy of probe selection becomes critical.

There are several published approaches and algorithms [24], [25], [26], [27], [28], [29], [30], [31] for the analysis of DNA sequences and selection of specific probes. Directly searching for probes based on traditional computational methods is labor-intensive and often requires much time. Probe selection could be simplified for the initial analysis of protein sequences that are more conservative. This approach was discussed in several communications [26], [31] but was not fully implemented because of the differences in amino acid and nucleotide sequences of the microorganisms. This approach could have a better chance of success for analyzing closely related DNA sequences such as cDNA of Influenza A and B viruses.

Considering the short length of HA and NA genes (1700 and 1400 nucleotides, respectively), finding a set of probes specific to each sub-type of HA and NA is not simple. First, there is a substantial similarity between sub-types of these proteins. Second, the cDNA structure of different sub-types of HA depends on the sub-type of NA present in the virus, and vice versa. This complicates the selection of probes specific to the entire sub-type regardless of the matching NA sub-type (e.g., H1N1, H1N2, … H1N9). In this study, we have explored the selection of probes based on amino acid sequences to create a universal microchip that could determine all sub-types of HA and NA of Influenza A virus.

## Results and Discussion

The selection of microarray probes was performed in two steps using custom software developed in our lab. During the first phase, amino acid sequences typical of the sub-type of interest, but absent from other sub-types, were identified. During the second phase, oligonucleotide probes were derived from these conserved peptides and their properties optimized. The rationale for this approach is that microorganism properties are determined mostly by amino acid sequences, rather than nucleotide sequences. In addition, amino acid sequences are more conserved, which simplifies the search for similar sequences.

**Figure 1:**
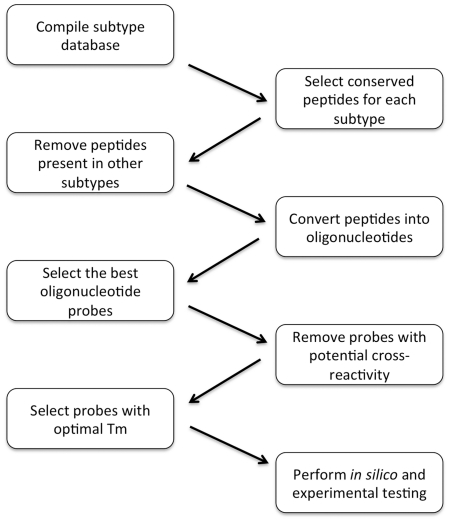
Scheme of the selection of oligonucleotide probes.

The process that will be illustrated using the example of the NA gene included the following steps (Fig. 1):

- Selection of nucleic sequences from GenBank and their translation into polypeptides
- Selection of peptides common for the NA sub-type of interest
- Removal of peptides present in other sub-types of NA
- Reverse translation of these peptides into nucleic acid probes
- Selection of the most representative probes
- Removal of oligonucleotide probes that can bind other NA sub-types after one or two mutations
- Selection of probes forming duplexes with target DNA with optimal melting temperatures

### Selection of conserved peptide sequences

Genomic sequence databases contain a number of incomplete sequences that complicate selection of universal probes. Therefore only protein sequences longer than 200 amino acids were analyzed. Conserved stretches of seven amino acids were identified among peptides from each sub-type of NA, regardless of a matching HA sub-type. This peptide length was chosen because using a longer stretch would significantly reduce the number of oligonucleotide probes typical for each sub-type, and shorter peptides would produce less specific probes. Because it was not possible to find peptides common to all proteins of the same sub-type (due to sequence variability and because some genes were not completely sequenced), the most common peptides that were present in more than 90%–95% of the proteins were selected.

Next, peptides unique for the serotype of NA were selected by removing peptides also found in other sub-types. Therefore, nine sets of peptides that were 7 amino acids in length identifying each sub-type of NA were selected.

The example below presents the amino acid sequence of the NA of A/California/UR06-0552/2007(H1N1) isolate. Peptides shown in bold italics are typical for 90% of N1 subtype neuraminidase, while absent from all other sub-types (N2–N9). There was a total of 89 such 7-mer peptides.

MNPNQKIITIGSISIAIGIISLMLQIGNIISIWASHSIQTGSQNHTGICNQRIITYENSTWVNHTYVSINNTNVVAGKDKTSVT***LAGNSSLC***SISGWAIYTKDNSIRIGSKGDVFVI***REPFISCSHLECRTFFLTQGALLNDKHSNGTVKDRSP***YRALMSCPL***GEAPSPYNS*** *K* ***FESVAWSASACHDG***MG***WLTIGISGPD***N***GAVAVLKYNGIIT***ETIKSWKKR***ILRTQESEC***VCVNGSCFTIMTDGPSNGAASYKIFKIEKGKVTKSIELNAPNF***HYEECSCYPD***TGTVM***CVCRDNWHGSNRPWVSFNQNL***D***YQIGYICSGVFGDNPR***PKDGKGSCNPVTVDGADGVKGFSY***KYGNGVWIGRTKS***NRLRK***GFEMIWDPNGWT***DTDSDFSVKQDVVAI***TDWSGYSGSFVQHPELTG***LDCI***RPCFWVEL***VRGLPRENA***TIWTSGSSISFCGV***DSDTAN***WSWPDGA***ELPFTIDK

### Selection of oligonucleotide probes

The conserved 7-mer peptides were reverse translated into the respective sets of 21-mer oligonucleotides. If peptides partially overlapped with a shift of one or two amino acids, then the corresponding oligonucleotide probes would produce largely redundant information about the sub-type of NA. In these cases only one such probe was selected using the following criteria. Let us consider peptide segment LAGNSSLC, represented by two 7-mer peptides: LAGNSSL and AGNSSLC. For these peptides, sets of 51 and 43 oligonucleotide probes were generated (Table 1). Selection of one of these two sets was made based on the following criteria:

1. Out of several sets of oligonucleotide probes sorted by the number of times each one was found in the set of analyzed sequences, the set in which the first probe was most represented was selected.
2. If the representation of the first probe was equal, then selection was based on the first two probes.
3. Again, if the representation was equal, the set with the minimal number of probes was selected.

**Table 1:** Oligonucleotide probes for N1 neuraminidase generated for LAGNSSL and AGNSSLC peptides.

#	LAGNSSL	#	AGNSSLC
1	TTAGCGGGCAATTCATCTCTT- (1043)*	1	GCGGGCAATTCATCTCTTTGC- (1022)
2	TTGGCCGGCAATTCATCTCTT- (708)	2	GCCGGCAATTCATCTCTTTGT- (769)
3	TTAGCCGGCAATTCATCTCTT- (121)	3	GCAGGCAATTCCTCTCTCTGT- (94)
4	CTAGCAGGCAATTCCTCTCTC- (98)	4	GCCGGCAATTCATCTCTTTGC- (76)
5	CTAGCCGGCAATTCCTCTCTT- (62)	5	GCCGGCAATTCCTCTCTTTGC- (69)
6	TTAGCGGGCAATTCCTCTCTC- (61)	6	GCGGGCAATTCCTCTCTCTGC- (67)
7	TTGGCAGGCAATTCGTCTCTT- (37)	7	GCGGGCAATTCATCTCTTTGT- (41)
	..............................		..............................
42	CTAGCAGGTAATTCCTCTCTT- (1)	42	GCGGGTAATTCCTCTCTCTGC- (1)
43	CTAGCAGGCAATTCTTCTCTC- (1)	43	GCGGGAAATTCATCTCTTTGC- (1)
44	CTAGCAGGCAACTCCTCTCTC- (1)		
45	TTGGCCGGTAATTCATCTCTT- (1)		
46	TTAGCCGGCAATTCATCTCTC- (1)		
47	CTAGCGGGCAACTCCTCTCTC- (1)		
48	TTGGCCGGCAATTCATCTCTC- (1)		
49	CTAGCGGGCAATTCATCTCTC- (1)		
50	CTAGCAGGCAATTCCTCTCTA- (1)		
51	TTAGCGGGAAATTCATCTCTT- (1)		

*The number of viral cDNA sequences containing each probe is given in brackets.

If all parameters in the above steps were equal, then the set was selected randomly; frequently, the first set was chosen.

In the above example (Table 1), peptide LAGNSSL, coded by 51 oligonucleotide sequences, met these criteria. Out of these oligonucleotide sequences, probe number 1 (TTAGCGGGCAATTCATCTCTT) was present in 1043 sequences of NA genes, while probe number 2 (TTGGCCGGCAATTCATCTCTT) was present in 708 out of the total 2696 analyzed NA sequences of this sub-type. For other probes, these numbers were significantly lower. If the continuous conserved peptide was long, as for REPFISCSHLECRTFFLTQGALLNDKHSNGTVKDRSP (see amino acid sequence of A/California/UR06-0552/2007[H1N1] isolate), several overlapping peptides shifted 3–7 amino acids relative to each other were selected to maximize the use of independent sequence information.

Principles used for the selection of probes for other sub-types of neuraminidase (N2–N9) were similar to the above example. Evidently, the number of oligonucleotide probes for the entire set of peptides can be very high, reaching several thousands. Many represent only one viral cDNA sequence (Table 1). Therefore, the next step was selection of the sub-set of an optimal size (20–24 probes), maximizing the chances of successful determination of NA sub-types. The simplest solution would be to choose only highly represented probes. However, the downside of this approach is that the procedure would be biased for probes specific to variants of NA from viruses containing the most prevalent sub-type of HA. Therefore we have used a special procedure to ensure more uniform representation of sequences.

### Reducing representation bias

The existing nucleotide databases predominantly contain sequences of NA gene of sub-types N1 and N2, while there is significantly less information about other sub-types. Similarly, the HA gene is mostly represented by sub-types H1, H3, and H5. In addition, within each sub-type of NA sequences of different matching sub-types of HA are also not represented equally (Tables S1 and S2). For instance, the N1 genotype is predominantly matched with sub-types H1 (1140 out of 2696 sequences or 42% of the database) and H5 (1295 or 48%), while some other HA sub-types were under-represented (H4, H10, H12) or absent altogether (H8, H13, H14, H15). The correlation between the structure of the NA gene and the type of HA is determined by coevolution of Influenza A viruses: changes in one gene are accompanied by changes in other genes [32], [33].

**Figure 2:**
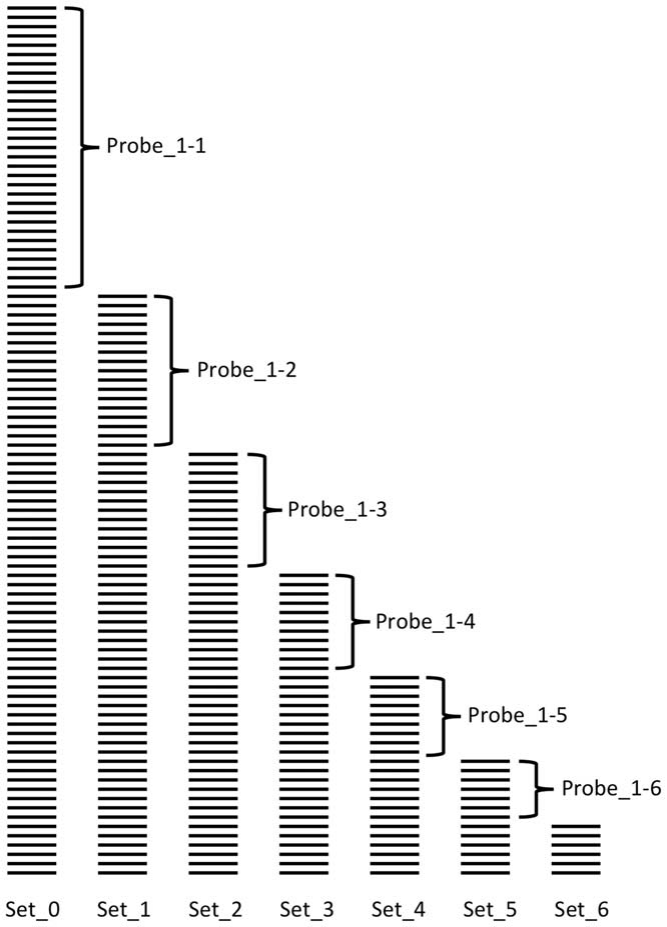
Selection of the most representative oligonucleotide probes.

To minimize the representation bias, the following approach was used (Fig. 2). First, the probe (Probe_1-1) most represented in all sequences (Seq_0) was selected from the entire set of probes (Probe_Total). Next, sequences containing Probe_1-1 were removed from Seq_0, producing a smaller set Seq_1. Next, Probe_1-2 that was represented most in Seq_1 was identified and removed from the set. This iterative operation was repeated to generate Probe_1-3, Probe_1-4, Probe_1-5, and Probe_1-6.

During the next phase, the first six probes (Probe_1-1–Probe_1-6) were removed from the entire pool of probes (Probe_Total), and the remaining probes were subjected to the same selection procedure using the entire starting DNA database Seq_0 to generate an additional set of probes Probe_2-1–Probe_2-6. The entire procedure was repeated three more times to create three additional sets of six probes. The structures of the resulting 30 probes are presented in Table S3.

If the number of sequences was small (as in the case of N4 neuraminidase represented by 31 cDNA sequences), the first generation probes Probe_101–Probe_1-3 completely determined the entire DNA database. In this case, to find more probes (20–24), iterations were repeated more than five times.

Table 2 shows that with an increased number of iterations, the number of highly represented probes drops from 1257 to 1107 (Probe_1-1 and Probe_5-1), while the number of poorly represented probes increases from 33 to 111 (Probe_1-6 and Probe_5-6).

**Table 2:** Sequences of oligonucleotide probes for sub-typing of N1 neuraminidase.

Probe_1-1	CAAGAGTCTGAATGTGCATGT	1257	1(2) N2
			13(2) N3
			2(2) N4
			1(1)+1(2) N6
			12(1)+10(2) N7
			2(2) N9
Probe_1-2	TTTGAGATGATTTGGGATCCT	922	1(1) N2
Probe_1-3	GGGAGTTTTGTCCAGCATCCA	183	1(0) N2
Probe_1-4***	GGTAATGGTGTTTGGATAGGA	91	8(2) N4
			1(0)+2(2) N2
Probe_1-5	ATAACAGACACTATCAAGAGT	54	1(0) N2
Probe_1-6***	GGCAATGGTGTTTGGATAGGA	33	8(2) N4
			1(1) N2
Probe_2-1	AAAGGGTTTTCATTTAAATAC	1143	1(1) N2
Probe_2-2	AGTGGGAGCAGCATTTCTTTT	827	1(0) N2
Probe_2-3***	GCAAGTGCTTGCCATGATGGC	242	5(2) N8
			1(0) N2
Probe_2-4	CACTATGAGGAATGCTCCTGT	164	1(2) N2
Probe_2-5	GCAAGTGCTTGTCATGATGGC	109	1(1)+1(2) N2
Probe_2-6***	TGCAGGGACAACTGGCATGGT	43	15(2) N7
			1(1) N2
Probe_3-1	ATAGGATATATATGCAGTGGA	1139	
Probe_3-2	TGGGTGTCTTTTAATCAAAAC	803	
Probe_3-3	GAGTGCTCCTGTTATCCTGAT	219	1(1)+1(2) N2
Probe_3-4	AAATACGGCAATGGTGTTTGG	129	1(1) N2
Probe_3-5	GGGTACATATGCAGTGGGGTT	101	1(2) N2
Probe_3-6	AGACCTTGCTTCTGGGTTGAA	67	
Probe_4-1***	TGGTTGACAATTGGAATTTCT	1112	1(2) N4
			1(2) N2
Probe_4-2***	GAATGTTCCTGTTACCCAGAC	774	5(2) N5
Probe_4-3	TGCTCCTGTTATCCTGATGCT	225	1(1) N2
Probe_4-4***	TGCAGGGATAATTGGCATGGC	144	8(2) N7
			35(1)+16(2) N9
Probe_4-5	ATAGGGTACATATGCAGTGGG	102	
Probe_4-6	ATAGGATACATCTGCAGTGGG	84	
Probe_5-1	GGATATATATGCAGTGGAGTT	1107	
Probe_5-2	GGAAGTTTCGTTCAACATCCT	739	
Probe_5-3***	TTTGAAATGATTTGGGATCCA	210	1(2) N2
			3(2) N4
Probe_5-4	AATCGACCTTGGGTGTCTTTT	122	1(2) N2
Probe_5-5	AGTGGGAGCAGCATTTCCTTT	114	1(1) N2
Probe_5-6	CCATACAACTCAAGGTTTGAG	111	1(1) N2

*The total number of cDNA sequences in this analysis was 2623.

**The number of point substitutions in each probe is given in brackets. For instance, “1(0) N2” is a perfect probe for N9 neuraminidase; “35(1)+16(2) N9” means that 35 and 16 sequences of N9 neuraminidase contain oligonucleotide probe that differs from the current probe by one or two point substitutions, respectively.

***Probes that were not used in further experiments.

In addition, there are other ways to select probes. For instance, to include the least represented sequences (such as H8N1 and H10N1), the above method could be modified to exclude from Seq_0 those sequences that match more probes than a pre-set limit (N_thresh). By varying this parameter, one can change the share of probes specific to N1 neuraminidase represented by a small number of DNA sequences. At low N_thresh, the higher number of DNA sequences from the entire set will match at least N_thresh probes. In contrast, at high N_thresh, probe selection will be done essentially as in the first method. In the present example, for all remaining NA sub-types, sets of 30 probes were selected.

### Minimizing cross-subtype hybridization and probe optimization

To reduce possible false positive hybridization results, oligonucleotide probes were tested for matches to other sub-types after introduction of one or two nucleotide changes. Table S3 shows the primary set of probes for the determination of N1 neuraminidase. Some of these probes demonstrated potential matches with other sub-types (N2, N3, … N9) with one or two mutations. Therefore, they were excluded from the set.

It should be noted that even some perfect probes, as well as probes with one or two mutations, that recognize N1 neuraminidase genes can be found in sequences of N2, albeit very rarely (Table 2). Detailed analysis shows that the only sequence of N2 neuraminidase that binds these N1-specific probes is virus isolate Influenza A/mallard duck/Minnesota/1979, classified as H3N2. This sequence does not have any probes (perfect or mismatched by 1 or 2 nucleotides) specific to N2 neuraminidase. On the other hand, the sequence contains four perfect probes specific to N1. A similar situation exists for isolates of Influenza A/Equine/New Market/79 (published as belonging to N3 sub-type but matching N8 based on oligonucleotide probes pattern), A/ruddy turnstone/Delaware Bay/262/2006(H7N5) (N5 based on traditional typing, but N3 based on oligonucleotide probes), and A/Anas plathyrhynchos/Spain/1252/2007(H6N4) (N4 vs. N5).

**Figure 3:**
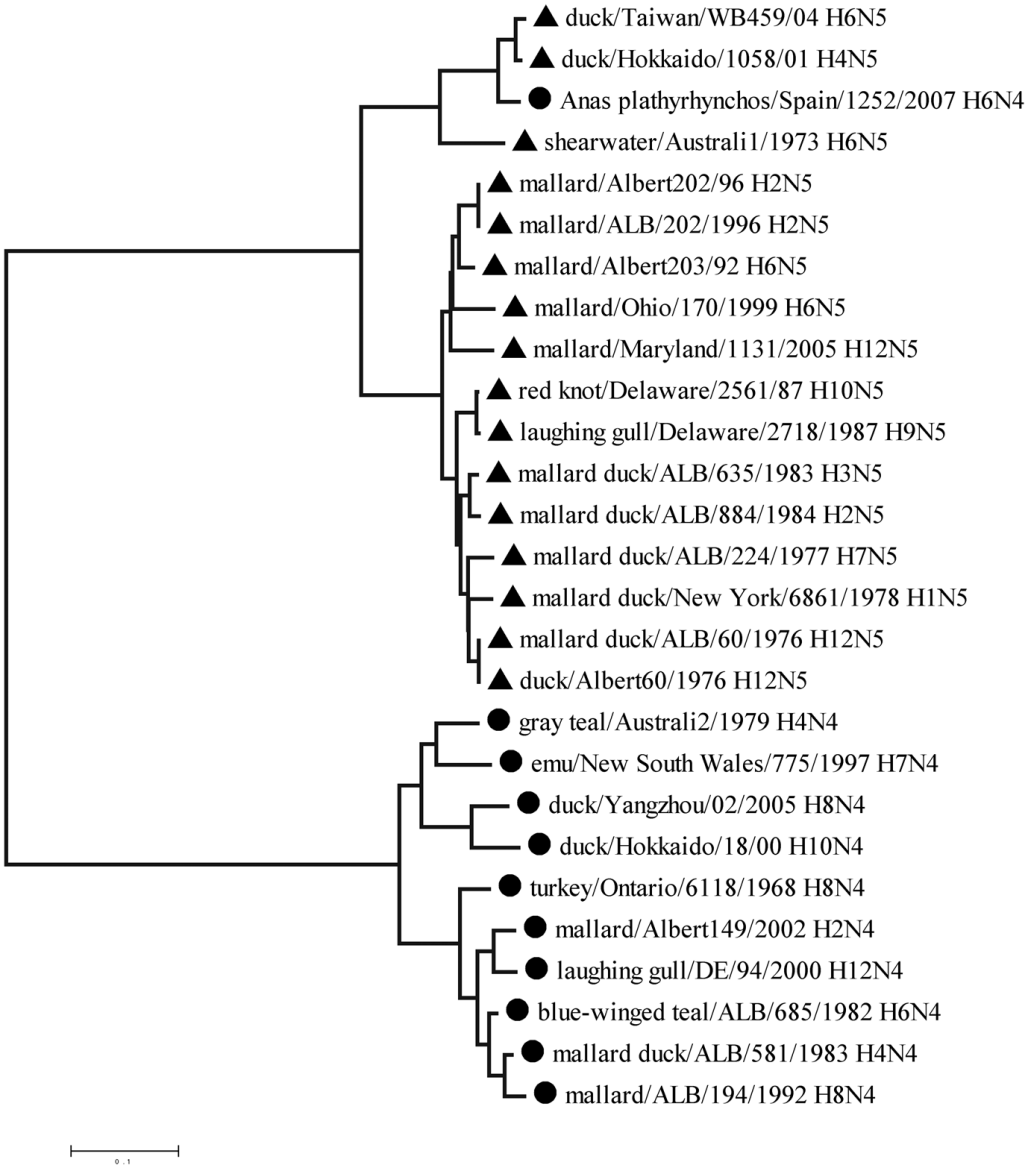
Phylogenetic tree for cDNA sequences from N4 and N5 neuraminidase isolates.

It appears likely that these four examples represent erroneous typing by conventional methods. Without access to the stocks of these viruses, it was impossible to directly test this hypothesis. However, independent phylogenetic analysis confirms that this might be the case. Fig. 3 shows the phylogenetic tree of a number of DNA sequences of N4 and N5 neuraminidase in which the A/Anas plathyrhynchos/Spain/1252/2007 isolate is located within the N5 clade.

Finally, to generate probes that would hybridize with their targets with uniform efficiency their lengths were modified to adjust the melting temperature of probe-DNA complexes. The range of melting temperatures was not very restrictive (10°C), and probe length adjustment included shortening or lengthening of a probe by 1–2 nucleotides within constant parts of codons.

### In silico testing of oligonucleotide probes

Length-modified probes were tested for matching sequences of other sub-types. Results showed that such modifications did not lead to the loss of specificity. By using the above procedure, probes specific to all nine sub-types of neuraminidase were selected (Table S3). The number of probes specific to each sub-type varied between 19 and 24; a total of 191 probes were selected.

A similar procedure was used for selection of probes to HA genes, with the exception of H14 for which there were only two sequences in GenBank (Table S4).

It is noteworthy that both amino acid sequences and primary DNA structures of the HA gene of H1N1 swine influenza virus substantially differed from other variants of H1 hemagglutinin. Therefore, none of the probes selected based on GenBank sequences deposited prior to March of 2008 matched the DNA of H1N1 swine influenza. This allowed us to design a set of probes for selective determination of H1 hemagglutinin of swine H1N1 influenza virus. Structures of these probes are shown in 4 (H1_swine). There are no significant differences in neuraminidase of this virus, and probes identified for N1 sub-type (Table S3) are suitable for genotyping the NA gene of swine H1N1 virus.

It is important to be able to use the probes for genotyping sub-types of HA and NA of viruses deposited in GenBank after March 2008 (set 1), i.e., DNA sequences that were not used to generate probes. Table S5 shows results of in silico testing for N1 neuraminidase in sequences deposited from March 2009–March 2010 (set 2). To minimize bias, only sequences of 1000 nucleotides or longer were used. The results show that the number of probes specific to N1 depends on the type of accompanying hemagglutinin. DNA of well-represented subtypes (H1N1 and H5N1) on average can be determined by 4–5 perfect probes, for both sets 1 and 2. For other sub-types of HA that are less represented, the average number of perfect probes vary from 2.3 to 5.6, and in most cases does not depend on the analyzed set of sequences. Similar conclusions can be made for other sub-types of NA and HA. This finding suggests that the probes developed in this work could be used for the genotyping of NA and HA sub-types of newly isolated strains of Influenza A. On the other hand, this result does not mean that the probe sets listed in Tables S3 and S4 are rigidly determined and cannot be expanded or modified. It may be useful to perform similar analyses in the future as the number of known sequences expands, closing gaps in the representation of some sub-types that exist today.

### Microarray hybridization

**Figure 4:**
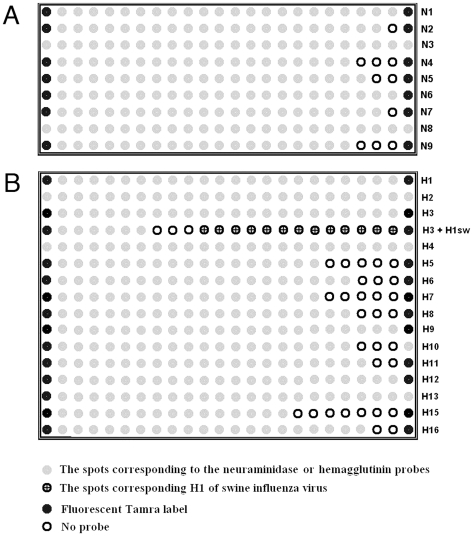
Microarrays layout. A, 24×9 spot microarray for sub-typing neuraminidase; B, 24×16 spot microarray for sub-typing hemagglutinin. Each line contains probes specific to individual sub-types of neuraminidase and hemagglutinin. Line number 4 in panel B contains probes specific to H3 and H1 of swine influenza virus.

The probes developed in this work were used to create a microchip containing two sub-arrays. One 16×9 spot sub-array contained NA-specific probes, while another 16×24 spot sub-array contained HA-specific probes (Fig. 4a and 4b). Probes contained 3′-amino hexyl linker to enable immobilization on glass slides modified with phenyl di-isothyocyanate [34]. Microchip edges were marked with (dT)_8_ oligonucleotide with 3′-amino hexyl linker and 5′-bound fluorescent dye TAMRA.

Microarrays were hybridized with amplicons prepared from cDNA of Influenza A virus. Because probes were selected from information regarding full-length sequences of NA and HA, it was important to generate PCR amplicons of appropriate length. To do this, multiplex PCR was used to amplify full-length HA and NA genes that were then used to generate fluorescently labeled DNA for hybridization with microarrays.

Multiplex PCR amplification of HA and NA genes of Influenza A virus was based on primers NA_F1, NA_R2, HA_F1, and HA_R2 described in [5] that were trimmed at the 5′-end. To increase specificity and the yield of fluorescently labeled DNA for hybridization, additional internal primers were selected to split each amplicon into two segments (see Materials and Methods).

Because of the significant sequence diversity, selection of internal PCR primers was performed in two steps. First, a conserved 7-mer peptide was chosen and reverse translated into a degenerate nucleotide sequence corresponding to a set of 21-base long oligonucleotides coding for this peptide. Amplification was conducted asymmetrically to generate predominantly single-stranded DNA for subsequent microchip hybridization.

The microchip created in this work was tested using samples representing different sub-types of hemagglutinin and neuraminidase (Table 3). A total of 40 samples of hemagglutinin genes (H1, H2, H3, H4, H5, H6, H7, H9, H10, H11, and H13) and 40 samples of neuraminidase (N1, N2, N3, N6, N7, N8) were tested. The hybridization of fluorescently labeled amplicons was performed at 55–60°C for 2 hours. Microchip slides were then washed, scanned, and analyzed using ScanArray software. To assess results of microarray genotyping, two parameters were used:

**Table 3:** Isolates of Influenza A virus used in this work and results of microarray genotyping.

№	Isolate	Virus sub-type	Taxon	HA		NA	
				Accession Number §	Successful genotyping call	Accession Number §	Successful genotyping call
1	A/goose/Krasnoozerka/627/2005*	H5N1	434196	DQ676840	(+)	EF205171	(+)
2	A/duck/Tuva/01/06*	H5N1	399313	DQ861291	(+)	DQ861293	(+)
3	A/turkey/Suzdalka/12/05*	H5N1	434198	EF205158	(+)	EF205172	(+)
4	A/Anas platyrhynchos/Chany Lake/9/03*	H5N3	326856	DQ007623	(+)	N/A	(+)
5	A/Chicken/Novosibirsk region/326/05*	H2N2	N/A	N/A	(+/−)	N/A	(+)
6	A/Domestic Duck/Novosibirsk region/41/05*	H4N6	N/A	N/A	(+)	N/A	(+)
7	A/Mallard/Mongolia/2307/2006*	H1N1	N/A	N/A	(−)	N/A	(+)
8	A/Solomon Islands/3/2006*	H1N1	464622	EU124137	(+)	EU124136	(+)
9	A/Irkutsk/165*	H6N1	N/A	N/A	(+)	N/A	(+)
10	A/Altay/1324/2007*	H3N8	N/A	N/A	(+)	N/A	(+)
11	A/mallard/Irkutsk/170/08*	H6N1	N/A	N/A	(+)	N/A	(+)
12	A/Pochard/Siberia/249/08*	H10N7	N/A	N/A	(+)	N/A	(+)
13	A/herring gull/Mongolia/454/08*	H13N8	N/A	N/A	(+)	N/A	(+/−)
14	A/pintail/Kamchatka/411/2007*	H7N1	N/A	N/A	(+)	N/A	(−)
15	A/mallard/Crimea/2027/2006*	H7N?	N/A	N/A	(−)	N/A	(+) N7
16	A/duck/Primorie/3691/02**	H12N2	397550	DQ787811	(+/−)	N/A	(+) N5
17	A/teal/Primorie/3628/02**	H9N2	397546	DQ787797	(+)	N/A	(+)
18	A/Moscow/IIV04/2009**	H1N1 swine	660123	GQ392022	(+)	GQ392023	(+)
19	A/Moscow/IIV05/2009**	H1N1 swine	667050	GQ494354	(+)	GQ494353	(+)
20	H10N7**	H10N7	N/A	N/A	(+)	N/A	(+)
21	H11N6**	H11N6	N/A	N/A	(+)	N/A	(+)
22	A/New Caledonia/20/91vr 116	H1N1	N/A	N/A	(+)	N/A	(+)
23	A/New Caledonia/20/1999	H1N1	381512	CY033622	(+)	CY033624	(+)
24	A/Texas/36/91	H1N1	380964	CY009316	(+)	CY009318	(+)
25	A/Texas/36/91 X-113	H1N1	380964	CY033655	(+)	CY033600	(+)
26	A/Solomon Islands/3/06	H1N1	464623	CY031340	(+)		(+)
27	A/Solomon Islands/3/06 Ivr-145	H1N1	N/A	N/A	(+)	N/A	(+)
28	A/St. Petersburg/08/2006 X-163	H1N1	461815	EU100725	(+)	N/A	(+)
29	A/St. Petersburg/08/2006 X-163B	H1N1	461817	EU100727	(+)	N/A	(+)
30	A/Sydney/05/97	H3N2	587884	AF087709	(+)	N/A	(+)
31	A/Sydney/05/97 Ivr-108	H3N2	N/A	N/A	(+)	N/A	(+)
32	A/Wisconsin/67/05	H3N2	393902	CY034118	(+)	CY034116	(+)
33	A/Wisconsin/67/05×Pr/8 X-161	H3N2	N/A	N/A	(+)	N/A	(−)
34	A/Wy/03/03×A/Pr/8/34	H3N2	N/A	N/A	(+)	N/A	(+)
35	A/Wyoming/03/2003	H3N2	480024	EU501362	(+)	N/A	(+)
36	A/New York/55/04	H3N2	518981	CY033638	(+)	CY033640	(+)
37	A/Ny/55/04×Pr/8 X-157a	H3N2	N/A	N/A	(+)	N/A	(+)
38	A/Pr8/34	H1N1	211044	CY033577	(+)	CY033579	(+)
39	A/Wuhan/359/1995	H3N2	63106	EU501120	(+)	N/A	(+)
40	A/Wsn/1933	H1N1	382835	CY034132	(+)	CY034134	(+)

§ For some samples obtained from Vektor and D.I.Ivanovsky Institute virus collections GenBank Accession numbers are unavailable.

(+) – sub-type call was made by using two parameters: average fluorescence of a spot and fraction of positive spots. In cases of discrepancy with previously determined genotype the new genotype is indicated.

(+/−) – sub-type call was made by one parameter: average fluorescence of a spot.

(−) – sub-type could not be determined.

*– cDNA of the Influenza A virus provided by Dr. E.M. Shestopalova, Vektor, Novosibirsk.

**– cDNA of the Influenza A virus provided by Dr. A.G. Prilipov, D.I.Ivanovsky Institute of Virology, Moscow.

1. Average fluorescence of a spot (Yμ) equal to the total fluorescence of all spots specific to a sub-type divided by the number of such spots.
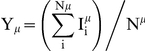
where 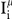 represents the fluorescence intensity of the i-th spot for sub-type μ, Nμ - the total number of spots of sub-type μ of NA or HA.

This parameter was chosen because the analyzed DNA could not only form perfect duplexes with probes, but also duplexes with one or two mismatches, providing additional information about the sub-type of test samples. For instance, for the A/Orenburg/IIV2974/2009(H1N1) isolate, 6 out of 22 probes can form a perfect duplex. For the A/Kyoto/07K303/2008(H1N1) isolate, the number of perfect duplexes is lower (2), but in this case there are also 7 duplexes with 1 mismatch. At the same time, the chances of forming stable complexes between these probes and other sub-types is substantially lower; the rules of probes selection allowed only three or more mismatches.

2. The fraction of positive spots, i.e., the number of spots specific to a sub-type with fluorescence exceeding the average fluorescence of all spots used for genotyping. Even though this parameter is linked to the first one, it identifies false positive results caused by high levels of fluorescence. Therefore, both parameters must be used for the evaluation of results. If both identify the same sub-type, this increases the reliability of this conclusion.

The error of calculation was determined based on variations in fluorescence intensity of marker spots located in the left and in the right columns of the microchip (Fig. 4). This error is integral and includes all errors related to fabrication, handling, and processing of the microchips.

**Figure 5:**
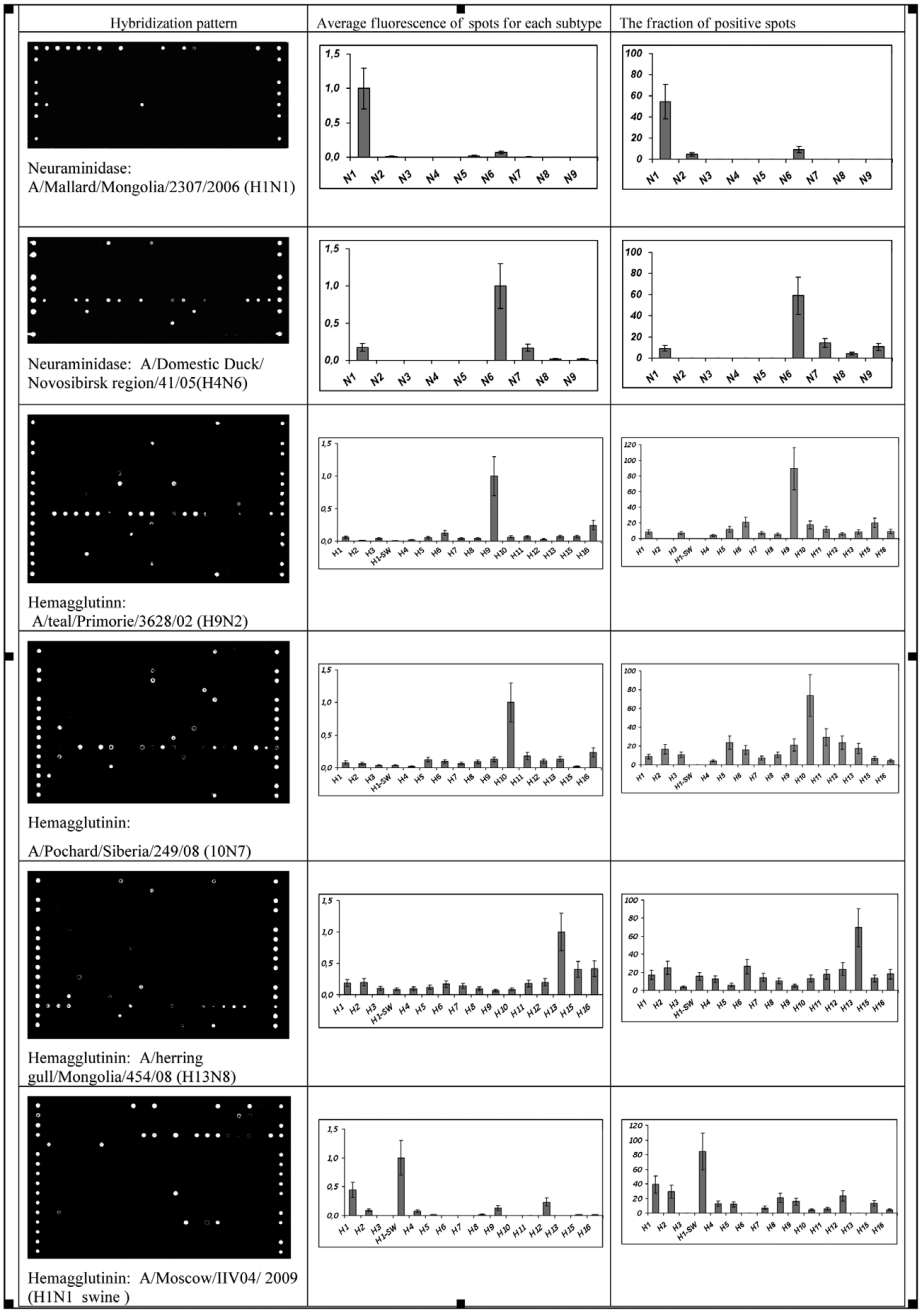
Microarray hybridization pattern obtained for amplicons of different sub-types of neuraminidase and hemagglutinin of the Influenza A virus and respective histograms showing average fluorescence of individual microchip elements. Hybridization was performed for 2 hours at 55°C.

Fig. 5 shows examples of hybridization results for amplicons of HA and NA of Influenza A viruses. It is clear that sub-types of the isolates can be identified unambiguously. For 36 out of 40 virus samples tested in this experiment, subtypes of HA and NA were clearly identified (Table 4). For one of the samples (A/mallard/Crimea/2027/2006), the subtype of NA was unknown but was clearly identified as N7. Another sample, A/duck/Primorie/3691/02, for which there was no NA data in GenBank, yet it was claimed to be H12N2, the microchip identified the NA as N5.

**Table 4:** Summary results of sub-typing of Influenza A isolates.

Sub-type	Total	(+)	(−)	(+/−)	Sub-type	Total	(+)	(−)	(+/−)
H1	12	11	1	-	N1	20	19	1	
H1_swine	2	2	-	-	N2*	12	10	1	1
H2	1	1	-	1	N3	1	1		
H3	10	10	-	-	N4	-			
H4	1	1	-	-	N5	-			
H5	4	4	-	-	N6	2	2		
H6	2	2	-	-	N7	2	2		
H7	2	1	1	-	N8	2	1		1
H8	-	1	-	-	N9	-			
H9	1	1	-	-	N?**	1	1		
H10	2	2	-	-					
H11	1	1	-	-					
H12	1			1					
H13	1	1	-	-					
Total	40	36	2	2		40	36	2	2

*One of isolates tests as N5.

**Sub-type of NA was unknown, but was determined to be N7.

(+) – sub-type call was made by using two parameters.

(+/−) – sub-type call was made by one parameter (average fluorescence of a spot).

(−) – sub-type could not be determined.

For two samples of HA from A/Chicken/Novosibirsk region/326/05 and A/duck/Primorie/3691/02 isolates and the NA from isolate A/herring gull/Mongolia/454/08, results were ambiguous, confirming the sub-type only by using one of the two parameters described above. We also could not determine the sub-type of NA for A/Wisconsin/67/05×Pr/8 X-161 and A/pintail/Kamchatka/411/2007 isolates, as well as the HA sub-type for isolates A/Mallard/Mongolia/2307/2006 and A/mallard/Crimea/2027/2006.

Interestingly, variations in temperature during hybridization do not affect hybridization patterns and relative distributions of intensities across the microchip. A temperature increase above 60°C reduces the number of unspecific spots but leads to the overall decrease of fluorescence, complicating the analysis and negatively impacting the overall performance of the test. Similarly, hybridization at temperatures below 50°C increases binding with non-specific oligonucleotide probes and leads to smaller differences between positive and negative spots. At the same time, the 10°C difference between Tm of different probes did not impact the performance of respective spots.

In conclusion, this communication describes a new method for the development of oligonucleotide hybridization probes that enables the creation of universal microchips for sub-typing HA and NA genes of Influenza A viruses. Evaluation of these probes, both in silico by matching them with database sequences and using them to experimentally identify new virus isolates, demonstrates high reliability of the proposed probes set for sub-typing HA and NA genes of Influenza A virus.

## Materials and Methods

Nucleotide sequences used in this work (Tables S1 and S2) were taken from GenBank and NCBI Influenza virus resource (http://www.ncbi.nlm.nih.gov, http://www.ncbi.nlm.nih.gov/genomes/FLU/FLU.html).

The selection of probes for sub-typing NA and HA genes was performed using custom software consisting of a set of Microsoft Basic macros that implements algorithms described above. Phylogenetic analysis was performed by version 4.0 of MEGA software using a neighbor-joining algorithm of cauterization.

Oligonucleotide probes and PCR primers were synthesized using ASM-800 DNA synthesizer (Biosset Ltd, Novosibirsk, Russia) using standard protocols. Microarrays were printed on glass phenyl isothiocyanate slides using Odyssey Calligrapher (BioRad) spotter with a 360 µm pin. Each slide contained four identical sub-arrays suitable for simultaneous hybridization of four samples. Each sub-array contained 9 and 16 lines of spots specific for NA and HA, respectively. The layout of the probes is shown in Fig. 4.

cDNA of samples 22–40 was produced from genomic RNA isolated from 140 µl of tissue culture fluid (virus titer 105–107 PFU/ml) using QIAquick Viral RNA Kit (QIAGEN, Valencia, CA). Reverse transcription was performed as follows. The mixture containing 1 µl of GACTAATACGACTCACTATAGGGAGCAAAAGCAGG primer (20 µM), 9 µl RNA extracted from 80 µl of virus, and 2 µl of the dNTP (10 mM) mixture was heated for 10 min at 65°C and chilled on ice. Next, 4 µl of 5× cDNA buffer, 1 µl water, and 1 µl of ThermoScript (Invitrogen, Carlsbad, CA) reverse transcriptase were added. The mixture was centrifuged and incubated for 5 min at 55°C and 55 min at 60°C.

To generate full-length genes of NA and HA, the following PCR primes were used:

NA_F1: 5′-CTATAGGGAGCAAAAGCAGGAGT-3′
NA_R2: 5′-CACTATAGAAGTAGAAACAAGGAGTTTTTT-3′
HA_F1: 5′-GACTCACTATAGGGAGCAAAAGCAGGGG-3′
HA_R1: 5′-GGTGACACTATAGAAGTAGAAACAAGGGTGTTTT-3′

The 50 µl of PCR mixture contained Qiagen PCR buffer (1.5 mM MgCl_2_, KCl, (NH_4_)_2_SO_4_, tris-HCl, pH 8.7), 1.5 mM MgCl_2_, 200 µM dATP, dCTP dGTP, dTTP, 1 µM of each primer, 1.25 U of Qiagen Hot Start Taq-polymerase, and 2 µl of template DNA solution. PCR was performed using iCycler (BioRad) with the described protocol: 15 min at 95°C followed by 42 cycles including 30 sec at 94°C, 30 sec at 58°C, and 5 min at 72°C.

To generate fluorescently labeled fragments, the following pairs of primers were used:

NA_F1: 5′-CTATAGGGAGCAAAAGCAGGAGT-3′
NA_R1: 5′-TAACAGGARCAYTCCTCRTARTG-3′
NA_F2: 5′-CAYTAYGAGGARTGYTCCTGTTA-3′
NA_R2: 5′-CACTATAGAAGTAGAAACAAGGAGTTTTTT-3′
HA_F1: 5′-GACTCACTATAGGGAGCAAAAGCAGGGG-3′
HA_R1: 5′-5′-TCWATRAANCCNGCDATDGCHCC-3′
HA_F2: 5′-GGDGCHATHGCNGGNTTYATWGA-3′
HA_R1: 5′-GGTGACACTATAGAAGTAGAAACAAGGGTGTTTT-3′

The amplicon lengths were ∼800 and ∼600 b.p. for neuraminidase and ∼1,100 and ∼700 b.p. for hemagglutinin. To generate predominantly single-stranded DNA for subsequent hybridization, asymmetric DNA was performed [12]. The composition of PCR mixture included (per 50 µl) Qiagen PCR buffer (1.5 mM MgCl2, KCl, (NH_4_)_2_SO_4_, tris-HCl, pH 8.7), 1.5 mM MgCl2, 200 µM dATP, dCTP dGTP, 70 µM dTTP, 20 µM dUTP-Cy3, 80 nM or forward primer, 1 µM of reverse primer, 1.25 U of Qiagen Hzot-Start Taq-polymerase, and 1 µl of full length amplicon template. PCR was performed in a BioRad iCycler under the following conditions: 15 min at 95°C followed by 34 cycles consisting of 30 sec at 94°C, 30 sec at 52–55°C, and 80 sec at 72°C. The amplification was monitored by electrophoresis in 1% agarose gel with EtBr staining. Fluorescently labeled amplicons were clarified from the excess of dNTPs using Qiagen gel filtration columns and were further dried in a vacuum centrifuge at 60°C.

The dried fluorescently labeled amplicons were reconstituted in 10 µl of water, supplemented with 10 µl of 2× hybridization buffer, heated for 2 min at 97°C, and chilled on ice before hybridization. 1× hybridization buffer contained 6× SSC, 5× Denhardt solution, and 0.1% Tween 20. Hybridization was performed in an ArrayIt chamber (Sunnyvale, CA) at 55–60°C for 2 hours. Next, slides were subsequently washed in 6× SSC, 2× SSC with 0.2 fi SDS, 2× SSC, and 1× SSC. Slides were scanned using Scan Array Express 2.0 (Perkin Elmer) at 543 nm (Cy3) and 633 nm (Cy5). The images were analyzed with ScanArray Express software (Perkin Elmer).

## Supporting Information

Table S1 **The number of isolates of Influenza A viruses with different sub-types of neuraminidase analyzed in this work.**
(XLS)

Table S2 **The number Influenza A virus isolates with different hemagglutinin sub-types tested in this work.**
(XLS)

Table S3 **Sequences of oligonucleotide probes for sub-typing of neuraminidase of Influenza A virus.** Oligonucleotide probes are shown in 5′- 3′ direction.
(XLS)

Table S4 **Sequences of oligonucleotide probes for sub-typing of Influenza A virus hemagglutinin.** Oligonucleotide probes are
(XLS)

Table S5 **Effectiveness of N1 neuraminidase sub-typing by using oligonucleotide probes.** Probes specific for N1 neuraminidase (see Table 5) and cDNA of neuraminidase longer than 1,000 nucleotides were used.
(XLS)
